# Prevalence, diagnosis, and disease course of pertussis in adults with acute cough: a prospective, observational study in primary care

**DOI:** 10.3399/bjgp15X686917

**Published:** 2015-09-28

**Authors:** Jolien Teepe, Berna DL Broekhuizen, Margareta Ieven, Katherine Loens, Kris Huygen, Mirjam Kretzschmar, Hester de Melker, Chris C Butler, Paul Little, Beth Stuart, Samuel Coenen, Herman Goossens, Theo JM Verheij

**Affiliations:** Julius Center for Health Sciences and Primary Care, University Medical Center Utrecht, Utrecht, the Netherlands.; Julius Center for Health Sciences and Primary Care, University Medical Center Utrecht, Utrecht, the Netherlands.; Laboratory of Medical Microbiology, Vaccine and Infectious Diseases Institute (VAXINFECTIO), University of Antwerp, Antwerp, Belgium.; Laboratory of Medical Microbiology, Vaccine and Infectious Diseases Institute (VAXINFECTIO), University of Antwerp, Antwerp, Belgium.; Scientific Institute of Public Health (WIV-ISP), Scientific Service Immunology, National Reference Centre *Bordetella*, Brussels, Belgium.; Julius Center for Health Sciences and Primary Care, University Medical Center Utrecht, Utrecht, the Netherlands.; Centre for Infectious Disease Control, National Institute for Public Health and the Environment (RIVM), Bilthoven, the Netherlands.; Nuffield Department of Primary Care Health Sciences, University of Oxford, Oxford, UK; Institute of Primary Care and Public Health, Cardiff University, Cardiff, UK.; University of Southampton Medical School, Primary Care Medical Group, Southampton, UK.; University of Southampton Medical School, Primary Care Medical Group, Southampton, UK.; Laboratory of Medical Microbiology, Vaccine and Infectious Diseases Institute (VAXINFECTIO), and Centre for General Practice, Primary and Interdisciplinary Care (ELIZA) Antwerp, University of Antwerp, Belgium.; Laboratory of Medical Microbiology, Vaccine and Infectious Diseases Institute (VAXINFECTIO), University of Antwerp, Antwerp, Belgium.; Julius Center for Health Sciences and Primary Care, University Medical Center Utrecht, Utrecht, the Netherlands.

**Keywords:** *Bordetella pertussis*, diagnosis, prevalence, primary care, prognosis, whooping cough

## Abstract

**Background:**

Most cases of adult pertussis probably remain undiagnosed.

**Aim:**

To explore the prevalence, diagnosis, and disease course of acute pertussis infection in adult patients presenting with acute cough.

**Design and setting:**

Prospective observational study between 2007 and 2010 in primary care in 12 European countries.

**Method:**

Adults presenting with acute cough (duration of ≤28 days) were included. *Bordetella pertussis* infection was determined by polymerase chain reaction (from nasopharyngeal flocked swabs and sputa) and by measurement of immunoglobulin G antibodies to pertussis toxin (PT) in venous blood at day 28. An antibody titre to PT of ≥125 IU/ml or PCR positive result in a respiratory sample defined recent infection. Patients completed a symptom diary for 28 days.

**Results:**

Serum and/or respiratory samples were obtained in 3074 patients. Three per cent (93/3074) had recent *B. pertussis* infection. Prior cough duration >2 weeks discriminated to some extent between those with and without pertussis (adjusted odds ratio 1.89, 95% confidence interval = 1.17 to 3.07; *P* = 0.010). Median cough duration after presentation was 17 and 12 days in patients with and without pertussis, respectively (*P* = 0.008). Patients with pertussis had longer duration of phlegm production (*P* = 0.010), shortness of breath (*P* = 0.037), disturbed sleep (*P* = 0.013) and interference with normal activities or work (*P* = 0.033) after presentation.

**Conclusion:**

Pertussis infection plays a limited role among adults presenting with acute cough in primary care, but GPs should acknowledge the possibility of pertussis in uncomplicated lower respiratory tract infection. As in children, pertussis also causes prolonged symptoms in adults. However, pertussis is difficult to discern from other acute cough syndromes in adults at first presentation.

## INTRODUCTION

Despite vaccination of children, pertussis remains endemic, largely because of waning immunity. The majority of pertussis cases have shifted from children to adults in countries where children are routinely vaccinated.^1^–^5^ Among unvaccinated infants pertussis remains most severe, sometimes even life threatening.^6^ Although the disease is, in general, milder among older children and adults, insight into the prevalence and disease course of pertussis in adults presenting with acute cough in primary care is not well described. Testing all patients presenting with cough for pertussis is not feasible. Distinguishing pertussis on clinical grounds alone from other causes of acute cough could help physicians better target testing for pertussis, prevent unnecessary further intervention, and help to set evidence-based expectations about disease course for patients.

Several international studies reported the proportion of pertussis in adults with cough in primary care,^1^,^7^–^10^ including Israel (*n* = 122),^7^ France (*n* = 217),^8^ the US (*n* = 212),^9^ and the UK (*n* = 145)^10^ reporting proportions of 7%, 32%, 13%, and 28%, respectively. However, these studies were small, used varying diagnostic criteria and recruited from a single country. Only one study has evaluated symptoms related to pertussis in adults with acute persistent cough in primary care. However, the evidence was limited because only 11 subjects out of a total of 156 had evidence of acute pertussis infection.^11^

Therefore, the present study evaluated the prevalence, diagnosis, and disease course of pertussis in adults presenting with acute cough in primary care between October 2007 and April 2010 in 12 European countries.

## METHOD

### Design and study population

This was a prospective study in primary care as part of the GRACE study (Genomics to combat Resistance against Antibiotics in Community-acquired lower respiratory tract infection [LRTI] in Europe; www.grace-lrti.org). GPs included 3104 patients from October 2007 to April 2010 in 16 primary care networks in 12 European countries (Belgium, England, France, Germany, Italy, the Netherlands, Poland, Spain, Slovakia, Slovenia, Sweden, and Wales). Eligible patients were aged ≥18 years who consulted their GP for the first time with an acute cough (duration of ≤28 days) as the main symptom, and were able to fill in study materials and provide written informed consent.^12^ Exclusion criteria were pregnancy, breast-feeding, and immunodeficiency. Additional for this analysis, patients without results on polymerase chain reaction (PCR) and/or serology were excluded. Ethical approval for the study was obtained in all participating countries.

**How this fits in**

In this study it was demonstrated that among adults presenting with acute cough in primary care acute pertussis infection does play a limited role, but GPs should acknowledge the possibility of pertussis in uncomplicated lower respiratory tract infection. As in children, pertussis also causes prolonged symptoms in adults. However, pertussis is difficult to discern from other acute cough syndromes in adults at the time of the first presentation.

### Measurements

Patients’ symptoms and comorbidities were reported on a standard case report form (CRF) on the day of presentation. At baseline, nasopharyngeal flocked swabs and, if available, sputa were taken and stored in the local laboratory until transport to the central lab in Antwerp for nucleic acid (NA) extraction by the NucliSENS^®^ EasyMag^®^ (Biomériux). NA extracts were analysed for *Bordetella pertussis* by real-time in-house PCR. At day 28, a serum sample was taken and analysed for immunoglobulin G antibodies to pertussis toxin (PT) (ESR 1201 G: Serion ELISA classic *Bordetella pertussis* toxin IgG, Virion/Serion).

Patients filled in a symptom diary until their symptoms had settled, to a maximum of 28 days. Among other things, they rated the severity of the following nine symptoms:

- cough;
- phlegm;
- shortness of breath;
- wheeze;
- chest pain;
- muscle aches;
- headache;
- disturbed sleep; and
- interference with normal activities or work.

Each symptom was scored from 0 to 6 (0 = no problem, 1 = very little problem, 2 = slight problem, 3 = moderately bad, 4 = bad, 5 = very bad, 6 = as bad as it could be). Review of patients’ notes (questionnaire in which GPs registered all contacts with patients for 4 weeks after the initial consultation) was performed to extract revisits to the GP with worsening symptoms, new symptoms, new signs, or illness necessitation admission to hospital within 4 weeks after the first consultation.

### Main outcomes

#### Prevalence of acute pertussis infection

This was defined as the number of pertussis cases as a proportion of the total number of included patients. For the proportion of pertussis per participating country, England and Wales were combined into ‘UK’.

#### Diagnosis of pertussis

Evidence of recent acute *B. pertussis* infection was defined as an antibody titre to PT of ≥125 IU/ml in convalescent serum and/or PCR positive result in a respiratory sample. The internationally used cut-off of 125 IU/ml has a sensitivity of 88% and specificity of 99%.^13^

#### Disease course of pertussis

Three outcomes were defined: duration of symptoms after the initial GP (index) consultation; unresolved symptoms at day 28; and worsening of illness defined as a re-consultation to the GP with new or worsening symptoms or signs, or hospitalisation within 28 days. The duration of symptoms after the index consultation was the number of days until the patient rated their symptoms as ‘no problem’ (0 in the symptom diary). Patients who were still experiencing symptoms at 28 days were treated as unresolved.

### Data analysis

#### Prevalence of pertussis

The prevalence of patients with evidence of acute pertussis infection and their 95% CI were calculated for patients with available PCR and/or respiratory samples.

#### Diagnosis of pertussis

Baseline characteristics were compared between patients with and without pertussis using the χ^2^ test for proportions and the Mann–Whitney U test for means. Patient symptoms at baseline were related to the presence or absence of pertussis. Based on the literature, the symptoms probably related to pertussis that were selected (age, prior cough duration >2 weeks, fever, disturbed sleep, wheeze, and interference with normal activities or work) were all included in the multivariable logistic regression analysis. This assessed which symptoms were independently associated with pertussis calculating odds ratios (ORs). Because the chance of having pertussis increases with longer cough duration, the association between prior cough duration > 2 weeks and pertussis was examined.^7^–^10^

#### Disease course of pertussis

Additional for the analysis of duration of symptoms, patients who did not return the diary were excluded. Duration of symptoms after the index consultation and unresolved symptoms at day 28 were compared per symptom between patients with and without pertussis using Cox regression and logistic regression, respectively. Logistic regression was used to compare ‘worsening of illness’ between those with and without pertussis. Statistical analyses were performed using SPSS (version 20.0) for Windows.

## RESULTS

### Prevalence of pertussis

A total of 3104 patients were recruited and a respiratory and/or serum sample was available for 3074. There was evidence of acute pertussis infections in 93/3074 adult patients (3%, 95% confidence interval [CI] = 2.5 to 3.7) presenting with acute cough in primary care. Seventeen patients were positive by both serology and PCR, 53 by serology only, and 57 by PCR only. The prevalence of pertussis varied across the 11 participating countries between 6.2% (6/97) in Sweden to 0% (0/80) in Italy (Table 1). The mean cough duration before the index consultation was 10 days (SD 8) in patients with pertussis and 9 days (SD 7) in those without pertussis (*P* = 0.008).

**Table 1.** Prevalence of pertussis cases by country

	***n/N***	**%**
Belgium	15/399	3.8
France	1/30	3.3
Germany	2/188	1.1
Italy	0/80	0.0
Netherlands	7/317	2.2
Poland	29/601	4.8
Slovakia	3/154	1.9
Slovenia	2/75	2.7
Spain	11/612	1.8
Sweden	6/97	6.2
UK	17/521	3.3
**Total**	**93/3074**	**3.0**

### Diagnostic value of symptoms

Table 2 shows the associations of symptoms at baseline with pertussis in patients with acute cough (*n* = 3074). Cough duration >2 weeks before the index consultation showed independent diagnostic value for pertussis (adjusted OR 1.89, 95% CI = 1.17 to 3.07; *P* = 0.010). Age, fever, disturbed sleep, wheeze, and interference with normal activities or work were not associated with pertussis.

**Table 2.** Association between diagnostic variables and pertussis in patients presenting with acute cough in primary care

	**All patients**	**Pertussis present**	**OR (95% CI)**			**Missing**
	***N*= 3074, *n* (%)**	***N*= 93, *n* (%)**	**Univariate**	**Multivariate**	***P*-value^a^**	***N* (%)**
Mean age, years (SD)	50 (17)	47 (16)	0.99 (0.98 to 1.00)	0.99 (0.98 to 1.00)	0.125	0 (0.0)
Male sex	1233 (40)	40 (43)	1.13 (0.75 to 1.72)	NA	–	0 (0.0)
Current smoker	860 (28)	25 (27)	0.96 (0.60 to 1.53)	NA	–	3 (0.1)
Comorbidity (pulmonary, cardiac, DM)^b^	847 (28)	24 (26)	0.91 (0.57 to 1.46)	NA	–	5 (0.2)
Cough duration >2 weeks before index consultation	525 (17)	25 (27)	1.79 (1.12 to 2.86)	1.89 (1.17 to 3.07)	0.010	45 (1.5)
Fever	1071 (35)	26 (28)	0.72 (0.45 to 1.14)	0.71 (0.44 to 1.14)	0.153	5 (0.2)
Disturbed sleep	1935 (63)	68 (73)	1.62 (1.02 to 2.57)	1.61 (1.00 to 2.60)	0.052	5 (0.2)
Wheeze	1309 (43)	35 (38)	0.81 (0.53 to 1.23)	0.79 (0.51 to 1.22)	0.281	5 (0.2)
Interference with normal activities or work	1937 (63)	63 (68)	1.24 (0.80 to 1.92)	1.28 (0.80 to 2.05)	0.311	3 (0.1)

*Data are presented as* n *(%), unless mentioned otherwise. DM = diabetes mellitus. NA = not analysed. OR = odds ratio. SD = standard deviation.*

a For multivariate analysis.

b *Pulmonary comorbidity = history of COPD, asthma, or other lung disease. Cardiac comorbidity = history of heart failure, ischaemic heart disease, or other heart disease. P*< *0.05 significant.*

### Disease course in patients with and without pertussis

Table 3 shows the duration of symptoms after the index consultation in patients with (*n* = 79) and without pertussis (*n* = 2437). Cough lasted a median of 17 days in patients with pertussis (interquartile range [IQR] 10–28) and 12 days in patients without pertussis (IQR 7–21) after the index consultation (hazard ratio 0.68, 95% CI = 0.51 to 0.91; *P* = 0.008). Patients with pertussis had longer duration of phlegm, shortness of breath, disturbed sleep, and interference with normal activities or work after the index consultation (Table 3). Cough, phlegm, shortness of breath, wheeze, disturbed sleep, and interference with normal activities or work were significantly more often unresolved at day 28 in patients with pertussis (Table 3). Patients with pertussis experienced significantly more new or worsened symptoms than those without pertussis, respectively 27% (25/93) versus 18% (521/2934) (OR 1.70, 95% CI = 1.07 to 2.72; *P* = 0.026).

**Table 3.** Duration of symptoms after the index consultation and unresolved symptoms at day 28 in patients with and without pertussis

	**Median duration of symptoms after the index consultation, days (IQR)**				**Unresolved symptoms at day 28, *N* (%)**				**Missing**
	**Pertussis present**	**Pertussis absent**	**Hazard ratio (95% CI)**	***P*-value**	**Pertussis present**	**Pertussis absent**	**Odds ratio (95% CI)**	***P*-value**	***N* (%)**
Cough	17 (10–28)	12 (7–21)	0.68 (0.51 to 0.91)	0.008	29/79 (37)	463/2431 (19)	2.47 (1.54 to 3.94)	<0.001	6 (0.2)
Phlegm	12 (5–26)	9 (5–17)	0.70 (0.53 to 0.92)	0.010	26/79 (33)	358/2424 (15)	2.83 (1.75 to 4.59)	<0.001	13 (0.5)
Shortness of breath	1 (0–11)	1 (0–5)	0.75 (0.57 to 0.98)	0.037	17/79 (22)	209/2417 (9)	2.90 (1.66 to 5.05)	<0.001	20 (0.8)
Wheeze	2 (0–10)	2 (0–7)	0.83 (0.65 to 1.06)	0.143	12/79 (15)	135/2419 (6)	3.03 (1.60 to 5.74)	0.001	18 (0.7)
Chest pain	1 (0–2)	1 (0–1)	0.99 (0.78 to 1.27)	0.962	5/79 (6)	98/2411 (4)	1.60 (0.63 to 4.03)	0.324	26 (1.0)
Muscle aches	1 (0–8)	3 (0–7)	0.91 (0.72 to 1.15)	0.417	6/79 (8)	126/2410 (5)	1.49 (0.64 to 3.49)	0.359	27 (1.1)
Headache	2 (0–8)	3 (0–7)	0.99 (0.78 to 1.25)	0.918	6/79 (8)	115/2416 (5)	1.65 (0.70 to 3.86)	0.253	21 (0.8)
Disturbed sleep	7 (2–14)	5 (1–9)	0.73 (0.57 to 0.94)	0.013	10/79 (13)	164/2416 (7)	1.99 (1.01 to 3.94)	0.048	21 (0.8)
Interference with normal activities or work	6 (1–12)	5 (1–9)	0.76 (0.60 to 0.98)	0.033	12/79 (15)	132/2413 (6)	3.10 (1.63 to 5.86)	0.001	24 (1.0)

*IQR = interquartile range. P* < *0.05 significant.*

## DISCUSSION

### Summary

In this European study 93/3074 (3%) adult patients presenting with acute cough in primary care had evidence of acute pertussis infection based on PCR analysis of respiratory samples and/or a serological test. Cough lasted longer after the index consultation in subjects with pertussis (17 days versus 12 days). At day 28 a considerable proportion of patients with pertussis still suffered from coughing (37%). In addition, duration of phlegm production, shortness of breath, disturbed sleep, and interference with normal activities or work lasted longer in patients with pertussis, and there was more often worsening of illness in those with pertussis (27% versus 18%).

The prevalence of pertussis varied across European countries and this could be because of diversity in vaccination schedules,^14^ type of pertussis vaccine, genetic variations of *B. pertussis,* and difference in help-seeking behaviour. Differences in vaccination coverage levels probably could not explain the differences because only three countries reported coverage levels below 95%.^15^ It was not possible to adjust for these factors.

### Strengths and limitations

To the authors’ knowledge this is the first large European prospective study on the prevalence, diagnosis, and course of pertussis in adults consulting primary care for acute cough. The broad inclusion criteria and recruiting from a wide range of European countries enhance the applicability of findings to usual care. In almost all adults (99%) a serum and/or respiratory sample were available. The samples were collected and analysed in the same laboratory with standardised procedures. Therefore it was possible to evaluate the prevalence of pertussis within primary care research networks in the participating countries in Europe. Data to calculate the outcome symptom duration after the index consultation were based on a diary reflecting subjective, patient-reported duration of symptoms. Study participants were recruited during 2.5 calendar years (October 2007 to April 2010) because seasonality of pertussis has been described in summer and autumn in the Netherlands and an epidemic occurs every 3 to 4 years.^16^,^17^ In 2007–2008 there was a small epidemic of pertussis in the Netherlands with an incidence of 5 per 10 000 inhabitants.^18^ To avoid overestimation the internationally accepted cut-off of 125 IU/ml in the convalescent serum sample was used to indicate recent pertussis infection.^13^

A limitation is that only patients with acute cough (≤28 days duration) were included. Therefore, the prevalence of pertussis in the complete domain of patients presenting with cough in primary care may have been underestimated because the *a priori* chance of pertussis is higher in patients with persistent cough.^1^,^7^–^10^ However, the aim was to assess whether it is possible to detect pertussis in an early stage, during the first consultation of acute cough to better target testing, prevent unnecessary further intervention, and improve patient education about disease course. It was not possible to report the full disease course for about one-third of the patients, because these patients still had symptoms at day 28.

Another possible limitation is that recent vaccination may have resulted in false-positive results. Vaccination status was not evaluated in this study, but it is expected that interference from vaccination-induced antibodies is low because only a few countries in Europe (Belgium, France, Germany, Sweden, and the UK) have adolescent, adult, or cocooning (booster vaccination of those with close contact with babies) vaccination strategies for pertussis.^3^,^14^ Moreover, antibody level decreases after vaccination (median half-life 17 months) so interference only occurs after recent vaccination.^19^

Finally, the power calculation of the GRACE study^12^ was not based on patients with pertussis and there is a risk of type 1 error (false-positive result) with multiple testing. Therefore the results should be treated with some caution.

### Comparison with existing literature

The prevalence of pertussis in adults with acute cough in this study is lower than the 7–32% reported in other studies with adult patients with cough.^1^,^7^–^10^ However, these studies included patients with persistent cough (often cough duration >3 weeks) in whom the prior probability of pertussis is higher. Moreover, the sensitivity and specificity of the diagnostic procedures varied between the studies. However, the prevalence of pertussis in patients with a cough duration >2 weeks was 5% (25/525) and was almost similar to a study in New Zealand that identified 7% (11/156) as having pertussis among adults (17–49 years) presenting with cough of 2 weeks duration in primary care.^11^ A study from Israel reported a pertussis prevalence of 7%, but they included only patients with acute respiratory infections with fever in primary care.^7^

### Implications for practice

Among adults presenting with acute cough in primary care acute pertussis infection does play a limited role, but GPs should acknowledge the possibility of pertussis in uncomplicated lower respiratory tract infection. As in children, pertussis also causes prolonged symptoms in adults. However, pertussis is difficult to distinguish from other acute cough syndromes in adults at the time of the first presentation.
